# Joint and Muscle Assessments of the Separate Effects of Botulinum NeuroToxin-A and Lower-Leg Casting in Children With Cerebral Palsy

**DOI:** 10.3389/fneur.2020.00210

**Published:** 2020-04-21

**Authors:** Nicky Peeters, Anja Van Campenhout, Britta Hanssen, Francesco Cenni, Simon-Henri Schless, Christine Van den Broeck, Kaat Desloovere, Lynn Bar-On

**Affiliations:** ^1^Department of Rehabilitation Sciences, KU Leuven, Leuven, Belgium; ^2^Department of Rehabilitation Sciences, University of Ghent, Ghent, Belgium; ^3^Department of Development and Regeneration, KU Leuven, Leuven, Belgium; ^4^Motion Analysis and Biofeedback Laboratory, Alyn Hospital, Jerusalem, Israel; ^5^Clinical Motion Analysis Laboratory, UZ Leuven, Pellenberg, Belgium; ^6^Department of Rehabilitation Medicine, Amsterdam UMC, Amsterdam Movement Sciences, Amsterdam, Netherlands

**Keywords:** cerebral palsy, tendon length, muscle length, spasticity, hyper-resistance, casting, Botulinum NeuroToxin, muscle stretch reflex

## Abstract

Botulinum NeuroToxin-A (BoNT-A) injections to the medial gastrocnemius (MG) and lower-leg casts are commonly combined to treat ankle equinus in children with spastic cerebral palsy (CP). However, the decomposed treatment effects on muscle or tendon structure, stretch reflexes, and joint are unknown. In this study, BoNT-A injections to the MG and casting of the lower legs were applied separately to gain insight into the working mechanisms of the isolated treatments on joint, muscle, and tendon levels. Thirty-one children with spastic CP (GMFCS I-III, age 7.4 ± 2.6 years) received either two weeks of lower-leg casts or MG BoNT-A injections. During full range of motion slow and fast passive ankle rotations, joint resistance and MG stretch reflexes were measured. MG muscle and tendon lengths were assessed at resting and at maximum dorsiflexion ankle angles using 3D-freehand ultrasound. Treatment effects were compared using non-parametric statistics. Associations between the effects on joint and muscle or tendon levels were performed using Spearman correlation coefficients (*p* < 0.05). Increased joint resistance, measured during slow ankle rotations, was not significantly reduced after either treatment. Additional joint resistance assessed during fast rotations only reduced in the BoNT-A group (−37.6%, *p* = 0.013, effect size = 0.47), accompanied by a reduction in MG stretch reflexes (−70.7%, *p* = 0.003, effect size = 0.56). BoNT-A increased the muscle length measured at the resting ankle angle (6.9%, *p* = 0.013, effect size = 0.53). Joint angles shifted toward greater dorsiflexion after casting (32.4%, *p* = 0.004, effect size = 0.56), accompanied by increases in tendon length (5.7%, *p* = 0.039, effect size = 0.57; *r* = 0.40). No associations between the changes in muscle or tendon lengths and the changes in the stretch reflexes were found. We conclude that intramuscular BoNT-A injections reduced stretch reflexes in the MG accompanied by an increase in resting muscle belly length, whereas casting resulted in increased dorsiflexion without any changes to the muscle length. This supports the need for further investigation on the effect of the combined treatments and the development of treatments that more effectively lengthen the muscle.

## Introduction

Cerebral palsy (CP), the most common childhood disability, is caused by an injury to the developing brain that occurs prior to, or shortly after, birth. In case of spastic CP, disturbed muscle innervation and biomechanical loading is combined with progressively worsening muscle contractures and joint hyper-resistance to movement. In the ankle joint, this hyper-resistance impairs the joint's passive range of motion (ROM), resulting in gait impairments such as equinus or excessive knee flexion (1).

Numerous studies have tried to establish the mechanisms that contribute to the development of ankle joint hyper-resistance in spastic CP. Recent literature indicates that hyperactive stretch reflexes, commonly labeled as spasticity, are considered to have less impact (2, 3). Instead, alterations such as reduced muscle volume and atrophy, cellular and genetic factors have been related to the increased joint resistance (4–6). Despite the likelihood of multifactorial contributions, the most common non-surgical treatment for joint hyper-resistance is intramuscular injection of Botulinum NeuroToxin-A (BoNT-A) which, mainly targets hyperactive stretch reflexes by causing temporary muscle paralysis. There is no known effect of BoNT-A in treating the non-neural components of joint hyper-resistance. The effectiveness of BoNT-A in correcting gait deviations, and passive joint ROM on the short-term, has been shown to improve when BoNT-A is combined with casting (7–9). The rational of this treatment combination is that paralysis, followed by passive immobilization in a neutral position, results in tissue elongation through physiological adaptation to prolonged stretch. However, few clinical studies reported the effects of BoNT-A treatment at the muscle level (10–18). Synthesis of existing clinical studies that include post-treatment assessment of muscle morphology is hindered by methodological differences between studies and lack of normalization to account for natural muscle growth. Therefore, results are inconclusive and whether the improved joint ROM occurs due to muscle remodeling or at the expense of other soft tissues, is not well-understood (19).

Given the frequent use of BoNT-A and casting in clinical practice, and the current uncertainties about their effect on muscle, more research is required. Knowledge of the working mechanism of the treatments will help understand how to combine them more efficiently. This will support the development of individually tailored treatments, where BoNT-A and casting are prescribed according to individually assessed causes of joint hyper-resistance. As a first step toward such patient-tailored medicine, careful assessment of the individual effects of BoNT-A and casting on different levels, ranging from joint to muscle and tendon, is required. The aim of the current study is to contribute to the understanding of the individual mechanisms by comprehensively evaluating the separate short-term effects of BoNT-A and lower-leg casting on ankle joint hyper-resistance, plantar flexor stretch reflexes, and muscle-tendon complex lengths. Additionally, the effects between the two treatments will be compared and the relations between treatment effects at the different levels are explored within each treatment group. It is hypothesized that BoNT-A will reduce hyperactive stretch reflexes, whereas casting will reduce the joint hyper-resistance by lengthening the muscle and tendon. Furthermore, any changes that occur at the joint level will be more related to alterations in the muscle and tendon lengths than to a reduction in stretch reflexes.

## Methods

### Participants

Children with spastic CP, aged between 4 and 17 years, were recruited from the Clinical Motion Laboratory of the University Hospital Pellenberg (Belgium), and included when the multidisciplinary clinical team concluded that there was an indication for BoNT-A treatment of the medial gastrocnemius (MG) and casting of their ankle joint. This decision was based on the results of routine clinical assessments of spasticity by means of Modified Ashworth scale (20) and Tardieu R1 angle (21), ROM (by means of goniometry), strength and selectivity (by means of manual muscle testing and clinical selectivity scores), and a 3D gait analysis. Furthermore, included children had a minimum of 20° ankle ROM in the most involved limb, no behavioral problems that would impede the ability to understand and perform the test procedure, and no dyskinesia, dystonic or ataxic features. Children who received BoNT-A injections in the calf muscles within 6 months prior to the first assessment, a Selective Dorsal Rhizotomy (SDR), or orthopedic surgery on the lower-leg, were excluded. Children with a Modified Ashworth Scale score of 2 or higher in the hamstrings and psoas muscles, indicating greater proximal compared to distal muscle involvement (and therefore not suited to receive ankle stretching casts without multilevel BoNT-A injections), were also excluded. The local University Hospitals' Ethics Committee (study number s57384) approved this study. All participants were informed on the content of the study and written informed consent for participation was obtained from all parents/legal guardians and children above the age of 12 years.

### Interventions

This investigation describes the first part of a larger study including a crossover design whereby BoNT-A (Botox® Allergan Ltd, Buckinghamshire, UK) and lower-leg casting were applied in two treatment sessions, rather than during the same session. The treatment sessions were separated by a two-week period. This approach allowed investigation of the individual effects of BoNT-A and casting at a group level, but also ensured that all patients eventually received both of their prescribed clinical treatments. Patients were randomly allocated by an independent researcher to one of two groups by minimization such that each allocation minimized imbalance between the groups across multiple factors including Gross Motor Functional Classification Scale (GMFCS) level, topographic classification (unilateral or bilateral involvement), age, and gender. Children allocated to the first group received BoNT-A in the MG as part of their multi-level treatment. BoNT-A dosage and muscle selection was based on patient weight, medical history, findings of a clinical examination, 3D gait analysis, and the clinician's experience. Injection was given under short masked anesthesia, and was ultrasound guided for visual identification of muscles and needle depth.

Children allocated to the second group received two weeks of lower-leg casting. Polyester casts were applied conform clinical guidelines by a specialized nurse, supervised by the treating orthopedic surgeon.

In the two weeks following treatment, children in both groups continued with their usual post-treatment physical therapy. Children allocated to the BoNT-A group continued their usual use of their ankle-foot orthoses.

### Assessments

Assessments were carried out on the most affected leg, defined by the most recent clinical examination. In the case of equal involvement, the left leg was assessed. Clinical examination of the plantar flexors including the Modified Ashworth Scale (20) and Tardieu R1 angle (21) as well as measurements of body weight and height were carried out before the treatment. Assessments of muscle and tendon lengths, ankle joint resistance and stretch reflexes were carried out before, and two weeks after, the individual treatments by the same experienced assessor.

#### Muscle and Tendon Lengths

Muscle and tendon lengths were assessed using 3D freehand ultrasound (3DfUS); combining conventional B-mode 2D ultrasound (Telemed EchoBlaster128, Vilnius, Lithuania) with 3D motion analysis (Optitrack NaturalPoint, USA), as previously described (22). The US acquisition parameters were kept constant between the different acquisitions (frequency, 10 MHz; depth, 5 cm; focus, 1.8–2.8 cm; gain, 46%; dynamic range, 44 dB and unaltered time-gain compensation) (23). This method has proven to be valid with a strong inter acquirer reliability in both healthy and pathological muscles (23, 24).

Subjects lay prone with the lower-leg supported by a small triangular cushion allowing ~20° of knee flexion and the ankle to rest comfortably over the edge of the cushion, to reduce bi-articular stretch on the plantar flexor muscles. This position is referred to as the resting ankle position. First, the MG was imaged at this resting ankle position. Secondly, imaging was carried out with the ankle at maximum dorsiflexion. Maximum dorsiflexion was achieved by a second examiner who manually fixated the ankle in this position while ensuring that maximal motion occurred at the subtalar joint, avoiding foot add-/abduction or pro-/supination. The US images were recorded by an experienced examiner starting from the medial femoral condyle until the most distal edge of the calcaneus. Two 3DfUS sweeps over the MG were carried out at each ankle position. The knee and ankle joint angles were measured with a goniometer at both positions.

#### Instrumented Assessment of Ankle Joint Resistance and Stretch Reflexes

A previously described assessment was used to distinguish between neural and non-neural components of ankle joint resistance during slow and fast passive joint rotations that stretched the plantar flexors (25). Participants were assessed supine with the lower-limb supported. Three inertial measurement units (IMU's) were used to track the movement of the upper-leg with respect to the lower-leg and the lower-leg with respect to foot. Calibration trials were carried out to define a known angular position and the direction of rotation. A six degrees-of-freedom torque load-cell (ATI mini45: Industrial Automation), attached to a foot orthosis, was used to move the ankle and measure the forces and moments applied to the ankle (25). Surface electromyography (EMG) data (Zerowire, Cometa, Milan, IT) were collected from the MG, lateral gastrocnemius, soleus and tibialis anterior. With the subject fully relaxed, three passive ankle rotations over the full ROM were performed, first at slow velocity (5 s to complete full ROM) and then as fast as possible. Between repeated rotations, there was at least a 7 s rest interval, to avoid post-activation depression (26).

### Data Processing

#### Muscle and Tendon Lengths

**Figure 1:**
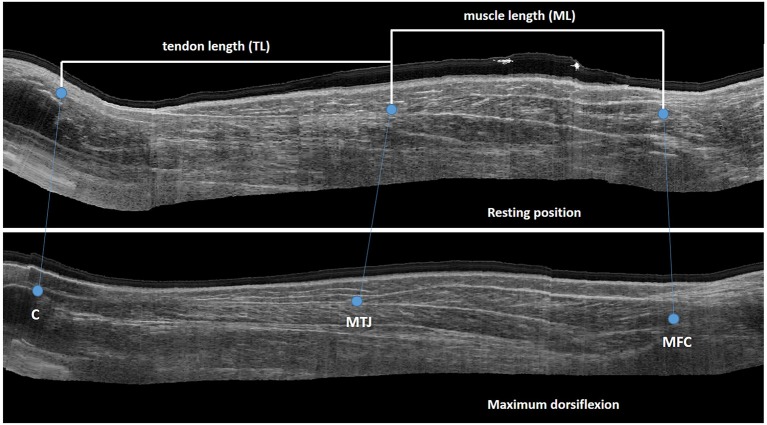
Sagittal US image with landmarks for extraction of muscle and tendon length *C, calcaneus; MTJ, muscle tendon junction; MFC, medial femoral condyle*.

An open-source software library, developed in Python, was used to create a 3-dimensional view of the MG by integrating 2D US data and motion tracking (22). The 3D reconstruction of the muscle-tendon complex was visualized in a custom-made workflow in Mevislab (www.mevislab.de) (22). Visualization in three planes allowed for accurate identification of the following anatomical landmarks: the most superficial part of the femoral condyle used to define the MG muscle origin; the muscle-tendon junction (MTJ), as the muscle insertion and tendon origin; and the most proximal point on the calcaneus as the tendon insertion. The lengths of the MG and corresponding tendon in the two ankle positions were extracted by calculating the Euclidean distances between these landmarks (Figure 1). Muscle-tendon complex length was calculated as the summation of muscle (ML) and tendon length (TL). The same assessor, who was blinded for group allocation and assessment session, extracted all lengths twice. Average lengths were calculated and used for the final statistical analysis. In addition, the standard error of measurement (SEM) associated with extracting the lengths was calculated from the square root of the mean square error from one-way ANOVA (27). The change in joint angle, ML and TL between the two joint positions was calculated and indicated the degree of the joint (Δ ankle angle) and muscle/tendon “extensibility.”

#### Instrumented Assessment of Ankle Joint Resistance and Stretch Reflexes

Data collected during the ankle joint resistance assessment were processed offline using a custom-made Matlab program (Mathworks, R2015a) as previously described (25). Raw EMG signals were filtered with a 6th order zero-phase Butterworth bandpass filter from 20 to 500Hz. The root mean square (rms) envelope of the EMG signal was defined by taking the square root after applying a low-pass 30 Hz 6th order zero-phase Butterworth filter on the squared raw signal. Joint angle and angular velocity were calculated from the IMU data by applying a Kalman filter (28). The net ankle joint moment was calculated from the forces and moments applied on the load-cell, the external moment arms, and the predicted torque caused by gravity on the orthotic (25).

ROM and maximum angular velocity were extracted from slow and fast passive ankle rotations. Average rms-EMG was calculated during an interval 200 ms before maximum velocity to 90% of the ROM, thereby emphasizing the velocity dependency of the hyperactive stretch reflex and excluding the effects of end ROM. To quantify the hyperactive stretch reflexes, average rms-EMG during slow velocity rotations was subtracted from average rms-EMG during fast rotations.

To quantify joint resistance, work during slow and fast passive rotations was defined as the average area underneath the torque-angle graph from maximum velocity to 90% ROM (25). Work during the slow passive rotation represented the non-neural component of joint resistance. The work during slow rotation was subtracted from that during fast rotation to calculate the neural component of joint resistance (25).

### Statistical Analysis

**Figure 2:**
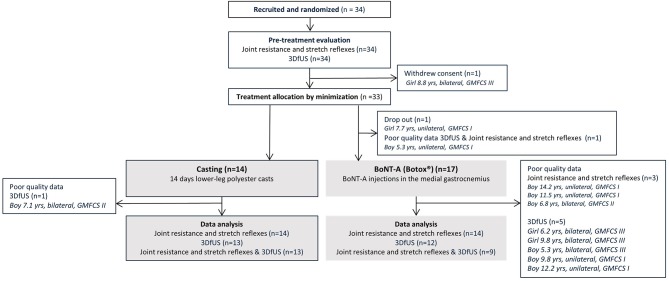
Overview of statistical analyses. *3DfUS, 3D freehand ultrasound; BoNT-A, Botulinum NeuroToxin-A*.

An overview of the used statistical analyses is included in Figure 2.

Statistical analyses were performed using SPSS statistics (version 25 IBM). Normality of data distribution was evaluated using the Kolmogorov-Smirnov test. Outlier analysis was performed visually. Within-group treatment effects were evaluated with Wilcoxon signed ranks tests. Between groups, the treatment-induced changes were compared using Mann-Whitney U-tests. In addition to being statistically significant, treatment-induced changes were only considered meaningful when larger than the SEM.

Associations between treatment-induced changes in ankle angles, muscle/tendon lengths, extensibility, ankle joint hyper-resistance and stretch reflex parameters were assessed by Spearman rank correlation coefficients for both treatment groups separately. Correlation values of 0.6 or higher were considered as strong (29).

Effect sizes were calculated according the formula: $r=Z/\sqrt{N}\;$ (30). The Z-score was extracted from the SPSS output of the Wilcoxon signed rank test. N represents the total number of observations. *R*-values of 0.5 or higher were considered as large effect sizes, whereas *r*-values above 0.3 were considered moderate.

## Results

**Figure 3:**
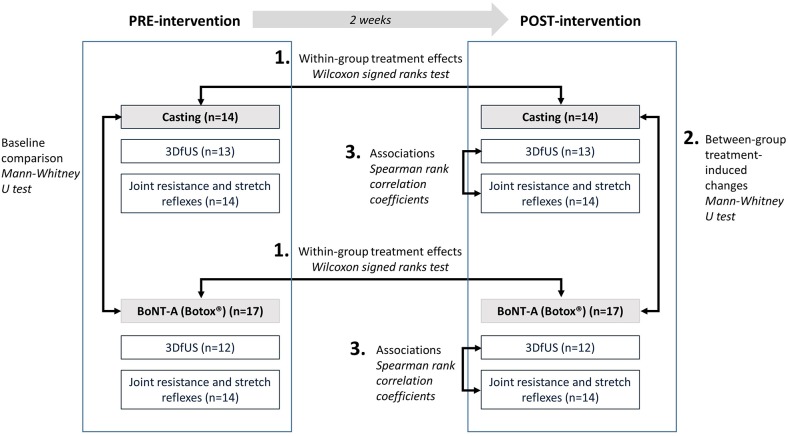
Flowchart of the inclusion of participants with an overview of the available and missing data. *3DfUS, 3D freehand ultrasound; GMFCS, gross motor functional classification scale; BoNT-A, Botulinum NeuroToxin-A*.

Thirty-one children with spastic CP (GMFCS I-III, age 7.4 ± 2.6 years) were included in this investigation. During the course of this study, some data were lost due to dropout and/or technical issues with measurement equipment. The flowchart of the patient enrolment and all available data that were included in the final data analyses, and subdivided per outcome, are presented in Figure 3. At baseline (Table 1), the two intervention groups did not differ in age, body weight, height, maximum dorsiflexion ankle angle, Modified Ashworth Scale, Tardieu R1 angle, the difference in ankle angle between resting and maximum dorsiflexion position (Δ ankle angle), extensibility of the muscle or tendon, muscle/tendon lengths, ROM, angular velocity, hyperactive stretch reflexes, and work. The number of included patients and their characteristics per sub-analysis are included in Supplementary Table 1.

**Table 1:** Participant characteristics.

	**Casting (*n* = 14)**	**BoNT-A (*n* = 17)**
Age (years)	7 (5–8.3)	7 (5.5–10.5)
Body weight (kg)	23.6 (18.4–28.7)	25 (19–27.6)
GMFCS level	I = 6, II = 6, III = 2	I = 11, II = 4, III = 2
Involvement	Unilateral = 7, Bilateral = 7	Unilateral = 12, Bilateral = 5
Gender	Male = 8, Female = 6	Male = 12, Female = 5
MAS^*^, knee extended	2 (1.5–3)	2 (1–3)
MTS R1^*^, knee extended (degrees)	−12.5 (−35 to −5)	−12.5 (−25 to 0)
Treatment details	2 weeks of lower-leg casting: *n* = 14 2 weeks of removable upper- leg casts worn during the night: *n* = 3	BoNT-A (Botox®) plantar flexors: 4 Units/kg (2.33–6.12) BoNT-A (Botox®) MG: 2.47 Units/kg (1.93–3.66) BoNT-A (Botox®) LG: 0.37 Units/kg (0.00–0.71) BoNT-A (Botox®) SOL: 1.49 Units/kg (0.00–2.04)
Use of day orthoses, prior to treatment	Frequently used (≥50% of the day) *n* = 11 Not frequently used (<50% of the day) *n* = 1 Not used *n* = 2	Frequently used (≥50% of the day) *n* = 14 Not frequently used (<50% of the day) *n* = 1 Not used *n* = 2
Use of night orthoses, prior to treatment	Frequently used (≥50% of the night) *n* = 2 Not frequently used (<50% of the night) *n* = 7 Not used *n* = 5	Frequently used (≥50% of the night) *n* = 2 Not frequently used (<50% of the night) *n* = 4 Not used *n* = 11

*Values are presented as medians with corresponding quartiles (p25–p75), or numbers (n)*.

* *median, minimum-maximum values. BoNT-A, Botulinum NeuroToxin-A; GMFCS, Gross Motor Functional Classification Scale; MAS, modified ashworth scale; MTS, modified tardieu scale; MG, medial gastrocnemius muscle; LG, lateral gastrocnemius muscle; SOL, soleus muscle*.

### Muscle and Tendon Lengths

**Figure 4:**
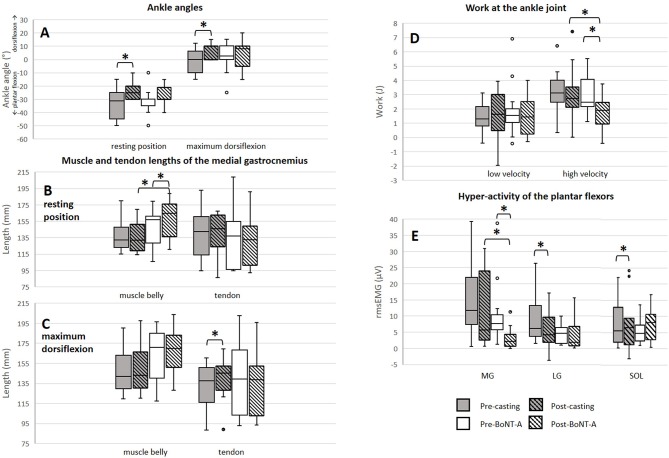
Results of muscle/tendon lengths, ankle joint resistance and stretch reflex assessments. **(A)** Change in ankle angles. **(B)** Muscle and tendon lengths at resting position. **(C)** Muscle and tendon lengths at maximum dorsiflexion. **(D)** Work at the ankle joint during slow and fast joint rotations. **(E)** Stretch reflexes of the plantar flexors during fast rotations. *significant results (p < *0.05, difference* > *SEM). mm, millimeter; J, Joule; rms EMG, root mean square of the electromyographic signal;* μ*V, microvolt; MG, medial gastrocnemius muscle; LG, lateral gastrocnemius muscle; SOL, soleus muscle; BoNT-A, Botulinum NeuroToxin-A*.

There was a positive significant treatment effect of casting on the resting (*p* = 0.004, pre median: −31.5°, inter quartile range (IQR): 20.0°; post median: −25.0°, IQR: 10.0°, effect size = 0.56) and maximum dorsiflexion (*p* = 0.026, pre median: 0.0°, IQR: 3.8°; post median: 10.0°, IQR: 10.0°, effect size = 0.44) angles (Figure 4). Knee angles remained constant between the assessments of muscle/tendon lengths before and after treatment. The SEM values for determining the muscle and tendon lengths were 2.25 and 2.54 mm, respectively. SEM values were found to be comparable to those previously reported (23). The absolute muscle and tendon lengths pre- and post-treatment are presented in Figure 4 and the treatment-induced change values are presented in Supplementary Table 2. Two weeks of casting caused a significant increase in the TL and muscle-tendon complex length at maximum dorsiflexion (5.6%, *p* = 0.039, effect size = 0.40, and 4.1%, *p* = 0.005, effect size = 0.63, respectively). These treatment-induced changes were also significantly larger than those found in the BoNT-A group (*p* = 0.002 and *p* = 0.001, respectively).

A significant increase in MG ML at the resting ankle position was found after BoNT-A injections (6.9%, *p* = 0.013, effect size = 0.53). Additionally, a significant increase in the TL at maximum dorsiflexion occurred, however this increase was within the range of the SEM. At the resting ankle position, ML after BoNT-A injections and the treatment-induced change in ML were significantly larger compared to those assessed following casting (*p* = 0.023 and *p* = 0.036, respectively).

Post-treatment, the extensibility of the ML and of the muscle-tendon complex length were significantly reduced after BoNT-A injections (−34.1%, *p* = 0.010, effect size = 0.55 and −36.3%, *p* = 0.013, effect size = 0.56, respectively), but unaltered by casting.

### Instrumented Assessment of Ankle Joint Resistance and Stretch Reflexes

The ankle joint ROM and angular velocities applied during passive joint rotation remained unchanged after either treatment. Effects of the treatments on plantar flexor stretch reflexes and joint work are displayed in Figure 4. Two weeks of casting caused a significant reduction in the stretch reflexes of the lateral gastrocnemius (−29.5%, *p* = 0.047, effect size = 0.44), and soleus (−17.5%, *p* = 0.034, effect size = 0.43). Post-casting, the average work assessed during slow, and fast minus slow stretch remained unchanged.

BoNT-A injections resulted in a significant reduction in the stretch reflexes of the MG (−72.4%, *p* = 0.003, effect size = 0.56) together with a reduction in work during fast minus slow stretch (−22.4%, *p* = 0.013, effect size = 0.47). There was a trend that this reduction in work was larger in the group receiving BoNT-A compared to casting (*p* = 0.050).

### Associations

Few correlations were found between the changes in joint angles, muscle/tendon lengths and ankle joint resistance. An overview of all correlation values and significance can be found in Supplementary Tables 4, 5.

In the casting group, there was a moderate positive correlation (*r* = 0.56, *p* = 0.049) between the change in TL at maximum dorsiflexion and the change in ankle angle (Δ ankle angle). Additionally, the increase in TL at maximum dorsiflexion was negatively related (*r* = −0.67, *p* = 0.050) to the reduction in stretch reflexes in the lateral gastrocnemius and the amount of work at high velocity (*r* = −0.60, *p* = 0.03). Furthermore, the increase in muscle-tendon complex length at rest was associated (*r* = −0.821, *p* = 0.02) with the reduction in stretch reflexes in the lateral gastrocnemius.

In the BoNT-A group, the reduction in hyperactive stretch reflexes of the soleus muscle was strongly correlated (*r* = 0.79, *p* = 0.036) to the increase in ML of the MG at rest. In addition, the reduction in work at fast minus slow velocity was strongly correlated (*r* = −0.77, *p* = 0.016) with the change in ML at the maximum dorsiflexion angle.

## Discussion

### Summary of Findings

The aim of this study was to gain more insight into the working mechanisms of BoNT-A and casting at the joint and muscle levels in children with spastic CP. Our hypotheses that BoNT-A targets the muscles' stretch reflexes, whereas casting targets the muscle belly and tendon length, were partly confirmed. Two weeks of casting increased the dorsiflexion angle, lowered the stretch reflexes in the lateral gastrocnemius and soleus muscles and increased the tendon length measured at maximum dorsiflexion position. BoNT-A reduced the stretch reflexes in the MG, reduced the joint resistance measured during fast passive ankle joint rotation, and increased the muscle length measured at the resting ankle position. Only a few associations between the treatment-induced alterations in joint angles, muscle or tendon lengths and measures of ankle joint resistance, and stretch reflexes were found. In summary, this study found rather minor changes at the muscle level after either intervention, with limited relationships between the effects at the joint, muscle or tendon lengths and stretch reflexes.

### Casting

We confirmed previous findings that maximum dorsiflexion increases after lower-leg casting (31, 32). However, since the joint angle at rest also moved toward greater dorsiflexion, the total joint ROM and change in ankle angle (Δ ankle angle) remained unchanged after treatment. While this result may make it easier to fit a foot into an ankle-foot orthosis configured toward dorsiflexion, it remains unclear to what extent this new biomechanical configuration is functionally useful. In the two subjects whose ROM did increase, this was mainly explained by an increase in their maximum dorsiflexion angle with an accompanied increased tendon length (Supplementary Figure 1). Interestingly, the three children who additionally received removable upper leg casts during the night showed an increase in muscle belly length at maximum dorsiflexion.

Since the plantar flexor muscles of children with spastic CP have been described as stiff and short (33), lengthening of these muscles through treatment is desirable. In clinical practice, it is thereby commonly hypothesized that an increase in dorsiflexion angle following casting is a result of lengthening the muscle. However, our results showed that the increase in maximum dorsiflexion angle post-casting was related to the increase in tendon length rather than alterations in muscle belly length. Hösl et al. (2015) reported increased tendon length following a period of ankle foot orthotic use (34) and Theis et al. (2013) reported similar results after a manual stretching intervention (35). As far as we are aware, our study is the first to report this effect following two weeks of casts.

Previous studies suggested that children with CP have a more compliant Achilles tendon (36, 37). In ambulant children with CP, this adaptation may be beneficial for achieving sufficient dorsiflexion during the stance phase of gait, but may compromise push-off power as the tendon may offer less resilience. Therefore, given that TL is already increased in CP, our findings highlight that caution has to be paid when applying passive stretches due to potentially enhancing the negative effects on the tendon. This is especially important when considering the coherence of the muscle-tendon unit as a complex and the efficiency of the muscle and tendon to interact during function. A recent study by Kalkman et al. (2019) concluded that increased tendon compliance in CP may result in reduced stretch stimulus to the muscle during ankle rotation, thus explaining the minimal effects of stretching interventions on muscle remodeling (38). In a follow-up study, they then confirmed that initially increasing tendon stiffness, by means of strength training, resulted in more efficient muscle stretch (38). Similarly, the group of Zhao et al. (2010) accomplished a reduction in Achilles tendon length and increased stiffness by combining passive stretching with active movement therapy (39). These findings suggest that stretching should be combined with strengthening exercises in order to optimally target the muscle belly.

The reduction in hyperactive stretch reflexes of the lateral gastrocnemius and soleus following casting was unexpected, but might be explained by the alteration in starting ankle angle, as the ankle was spontaneously more dorsiflexed after casting. Meinders et al. (1996) suggested that specifically at the ankle, stretch reflexes are reduced at a longer starting muscle-tendon complex lengths (40). Confirming this, we found that the reduction in hyperactive stretch reflex in the lateral gastrocnemius following casts was associated with an increase in tendon length.

### BoNT-A

BoNT-A targets the neural component of ankle joint resistance by reducing the hyperactive stretch reflexes. Reduction of hyperactive stretch reflexes is thought to create the opportunity for the muscle to act over a larger ROM and thereby improve its function (41). Our results confirmed the reduction of MG stretch reflexes, shown by a reduction in rms-EMG of the MG and in the reduced work needed to move the ankle joint during a high-velocity stretch of the plantar flexors. The question remains whether these changes additionally translate to morphological changes at the muscle level. Post BoNT-A, we observed an increase in the resting length of the MG muscle belly. Since spastic muscles have been described with a higher resting muscle tone (42), the increased MG muscle belly length may be a direct result of muscle tone reduction and the ability of the muscle to relax in this position. On the other hand, there were no changes to the muscle length measured at maximum dorsiflexion. Therefore, we found little evidence that the reduction in hyperactive stretch reflex of the MG post BoNT-A induced muscle remodeling. It is known that the muscle needs more time than the current short-term (two weeks) follow-up to adapt and no conclusions can be drawn about the medium- and long-term effect of BoNT-A on muscle length (43). Furthermore, muscle remodeling might take place after combining with casting.

### Combined Treatment

Answers regarding the combination of the treatments are currently being analyzed as the second part of this research project, which includes a crossover design with all the participants receiving the other treatment. In addition, this second part includes 3D gait analysis before and after receiving the two interventions allowing us to understand effects of the interventions on the functional level. Desloovere et al. (2007) showed that BoNT-A combined with casting was more effective in improving gait, compared to casting alone (44). However, it is unclear how these effects were achieved and additional insight, which we expect to gain with the second stage of this project, will likely facilitate the understanding of these findings. Following BoNT-A injections only, there were no changes in the ankle angles or joint ROM. Given the effects of casting on these parameters, there is reason to believe that the best results may be achieved when the two treatments are combined. Ideally, the changes seen on the muscle level following BoNT-A, in combination with static stretch as applied from casting, will result in the desired effect of increasing the joints ROM by promoting muscle length. Such an effect should result in greater ankle mobility that would be transferrable to the child's gait. However, given the finding that casting only increased the tendon length, very careful monitoring of how ankle mobility is achieved post-casting is essential, as it may be subject-specific. This highlights that an individualized and fine-tuned combination of the treatments is required. Furthermore, we can question whether a primarily passive stretch applied by casts is beneficial, as evidence shows that muscle activity is required to efficiently stretch the muscle and maintain an optimal configuration of the muscle-tendon complex (39).

Importantly, since neither treatment in isolation affected the increased joint resistance assessed during slow passive stretch, it is unlikely that the combination of the two treatments will affect resistance during slow stretch. If confirmed, this suggests that we need more effective treatments that address hyper-resistance over the full ROM, and not only increases the maximum dorsiflexion. If combined results are still not satisfactory, new treatment modalities should be developed to more efficiently target the muscle.

Our data showed a lack of strong responses on the group level. This reflects the large heterogeneity of the children with CP included in this study and therefore the need for larger samples. Given that the sample was taken from the clinical population seeking treatment for ankle joint hyper-resistance, this finding emphasizes the need to generate muscle-specific profiles and establish patient-tailored treatment. Furthermore, we highlight the importance of evaluating the underlying muscle and tendon structures rather than providing treatment based only on conclusions drawn from evaluations at the joint level.

### Limitations

The study has limitations that need to be acknowledged. This study investigated only the very short-term effects of the treatments. This allowed us to evaluate the separate effects without having to compromise on the children's clinical intervention plan. A study design in which the same study sample received both treatments including a wash-out period would have been methodically stronger. However, implementing such an investigation would differ too much from normal clinical treatment and would not be considered ethical (nor would including control groups from whom treatment was denied). In addition, considering the long time span of such a study, other parameters would influence the response.

This clinical study included a small dataset with missing data. As a result, not all participants were included in all analyses. When only including subjects who underwent both assessments (*n* = 23, BoNT-A *n* = 9, casting *n* = 13), most of our conclusions were still valid (Supplementary Table 3). Nevertheless, more studies need to be conducted to confirm the initial results of this study. Randomization by minimization was applied in order to minimize differences between groups in terms of pathology, age, body weight and height. Even though the groups did not perfectly match, there were no differences reported at baseline. Heterogeneity in treatment history and current treatment adherence are limitations when performing randomized controlled trials in children with CP. It is well-described that muscle morphology of children with CP is different from typically developing peers, although it remains unclear to what extent treatment history contributes to these alterations. This should be focus for further investigation. In addition, given the wide age range, and the effect of age on the ability of the muscle fiber to adapt, it is possible that the muscle fiber stage influenced our results.

The full data set of this investigation is published (DOI: https://doi.org/10.6084/m9.figshare.12009375.v1). When more data becomes available, sub analyses according to baseline characteristics may be carried out that will contribute to clinical implementation.

This study only investigated the muscle and tendon lengths of the MG as this is the muscle that is most frequently treated with BoNT-A in daily clinical practice. Future investigations with larger samples investigating more muscles are needed to confirm these initial findings. Given recent validation of the clinical applicability of the methods used in this study (23, 45), such investigations should be feasible in the near future.

We studied the alterations in the length of the entire muscle belly. Yet, this does not provide information on the fascicle behavior, especially since the MG is pennated. More-over, local fascicle remodeling in specific regions of the muscle may have occurred. Furthermore, since neither muscle length nor fascicle length necessarily represent sarcomere number or length, it is important to continue investigations that combine macro- and micro-structure responses to the applied treatments.

## Conclusion

This study presents initial findings on treatment response in a heterogeneous group of children with spastic CP. Intramuscular BoNT-A injections reduced the stretch reflex in the MG accompanied by an increase in resting muscle belly length, whereas casting resulted in increased dorsiflexion without any changes to the muscle length. These results indicate limited treatment-induced MG muscle remodeling. This supports the need for further investigation once the treatments are combined and for the development of treatments that more effectively lengthen the muscle belly. Additionally, future implementation of objective assessments will contribute to a better understanding of treatment effects, which could support clinical decision-making and help to identify responders.

## Data Availability Statement

The datasets generated for this study can be found in the Figshare repository DOI: https://doi.org/10.6084/m9.figshare.12009375.v1.

## Ethics Statement

The studies involving human participants were reviewed and approved by Ethical-Committee of the University Hospitals of Leuven/KU Leuven. Written informed consent to participate in this study was provided by the participants' parents/legal guardians.

## Author Contributions

This study was designed by LB, KD, and AV. NP, BH, and S-HS were responsible for data collection. FC and LB wrote the software for data analysis. AV evaluated eligibility of subjects. NP and LB conducted all presented analyses. All authors have had complete access to the study data throughout the study. NP, AV, BH, FC, S-HS, CV, KD, and LB contributed to the interpretation of the results and were involved in the critical revision and editing of the manuscript that was written by NP and LB. All authors approve the final version of the manuscript and agree to be accountable for the content of the work.

## Conflict of Interest

The authors declare that the research was conducted in the absence of any commercial or financial relationships that could be construed as a potential conflict of interest.
