# Supplementary material for: Joint and Muscle Assessments of the Separate Effects of Botulinum NeuroToxin-A and Lower-Leg Casting in Children With Cerebral Palsy
Source: Front Neurol. 2020 Apr 21;11:210. doi: 10.3389/fneur.2020.00210 (PMC7187925; doi:10.3389/fneur.2020.00210)
Supplement: Supplementary file 1 [file Data_Sheet_1.pdf]

Supplementary Material

Supplementary Table I. Patient characteristic per analysis. Values are medians and interquartile ranges.

	**Casting group**			**Botulinum NeuroToxin-A group**		
**Performed analyses**	Joint resistance and stretch reflexes (n=14)	Ultrasound (n=13)	Joint resistance and stretch reflexes + Ultrasound (n=13)	Joint resistance and stretch reflexes (n=14)	Ultrasound (n=12)	Joint resistance and stretch reflexes + Ultrasound (n=9)
**Age** (years)	7 (5 - 8.3)	7 (5-8,5)	7 (5-8,5)	7 (5-9.3)	7 (5.3-8.8)	7 (5-8.3)
**Body weight** (kg)	23.6 (18.4 – 28.7)	22.9 (18.4-28.4)	22.9 (18.4-28.4)	264.1 (18.7-28.2)	20.25 (17.6-26.9)	20.3 (17-27.2)
**GMFCS level**	I = 6, II = 6, III = 2	I = 6, II = 5, III = 2	I = 6, II = 5, III = 2	I = 9, II = 3, III = 2	I = 9, II = 3, III = 0	I = 8, II = 2, III = 0
**Uni- or bilateral involvement**	UL = 7<br>BL = 7	UL = 7<br>BL = 6	UL = 7<br>BL = 6	UL = 10<br>BL = 4	UL = 10<br>BL = 2	UL = 9<br>BL = 1
**Gender**	Male = 8<br>Female = 6	Male = 7<br>Female = 6	Male = 7<br>Female = 6	Male = 9<br>Female = 5	Male = 9<br>Female = 3	Male = 7<br>Female = 3
**MAS, knee extended***	2 (1.5-3)	2 (1.5-3)	2 (1.5-3)	2 (1-3)	1.5 (1-3)	1.5 (1-3)
**Tardieu R1*** (degrees)**, knee extended**	-12.5 (-35 - -5)	-10 (-35 - -5)	-10 (-35 - -5)	-15 (-25-0)	-15 (-25-0)	-15 (-25 – 0)

Values are presented as medians with corresponding quartiles (p25-p75), or numbers.
*median, min-max values.
GMFCS = Gross Motor Functional Classification Scale, MAS = Modified Ashworth scale MTS = Modified Tardieu Scale

Supplementary Table II. Median (and interquartile range) values of all outcome parameters
maxDF = maximum dorsiflexion, ML = muscle length, TL = tendon length, mm = millimeter, J = joule, rmsEMG = root mean square of the electromyographic signal MG = medial gastrocnemius muscle, LG = lateral gastrocnemius muscle, µV = microvolt, SOL = soleus muscle, ROM = range of motion

	**Casting (N=14)**			**Botulinum NeuroToxin-A (N=17)**			**POST comparison between groups**	**CHANGE comparison between groups**
Parameter	**PRE intervention** *median (p25-p75)*	**POST intervention** *median (p25-p75)*	**p-value**	**PRE intervention** *median (p25-p75)*	**POST intervention** *median (p25-p75)*	**p-value**	**p-value**	**p-value**
Ankle angle - resting (degrees)	-31.50 (-45.00--25.00)	-25.00 (-30.00--20.00)	*0.004*	-30 (-35 --30)	-30 (-30--21.25)	0.221	0.062	*0.015*
Ankle angle - maxDF (degrees)	0.00 (-10.00-6.25)	10.00 (0.00-10.00)	*0.026*	2.5 (0.00-10.00)	8.00 (-5.00-10.00)	0.414	0.413	0.220
Extensibility ankle angles (degrees)	35.00 (27.50-41.25)	30.00 (25.00-31.00)	0.121	35.00 (21.25-40.00)	30.00 (25.00-38.00)	0.782	0.555	0.350
ML - resting (mm)	132.00 (122.84-147.79)	132.18 (118.99-151.56)	0.959	156.50 (127.99-160.96)	164.96 (136.35-176.10)	*0.013*	*0.023*	*0.036*
ML - maxDF (mm)	141.88 (129.41-162.71)	143.16 (130.23-166.42)	0.139	170.67 (139.97-184.77)	170.16 (151.22-183.13)	0.388	0.069	0.821
Extensibility ML (maxDF-rest) (mm)	12.53 (9.40-16.15)	11.36 (5.76-21.21)	0.866	14.59 (7.45-25.02)	9.61 (4.80-14.85)	*0.010*	0.468	0.211
TL - resting (mm)	142.03 (113.54-160.24)	146.16 (123.86-162.45)	0.507	136.94 (95.80-154.26)	132.70 (101.22-149.40)	0.388	0.295	0.270
TL - maxDF (mm)	137.30 (116.09-150.82)	145.42 (128.41-152.40)	*0.039*	139.16 (103.08-168.12)	138.80 (102.98-152.38)	0.041	0.733	*0.002*
Extensibiliy TL (maxDF-rest) (mm)	.-4.35 (-12.30-2.48)	0.33 (-4.50-3.46)	0.196	2.15 (-2.44-15.62)	.-0.98 (-5.54-4.89)	0.213	0.776	0.093
Muscle-tendon complex - resting (mm)	284.33 (238.96-307.93)	278.78 (258.11-305.38)	0.203	290.97 (248.33-312.84)	306.87 (263.57-323.36)	*0.033*	0.651	1.000
Muscle-tendon complex - maxDF (mm)	271.78 (252.36-304.70)	287.66 (266.74-316.36)	*0.005*	302.57 (263.11-325.24)	300.96 (272.87-328.90)	0.110	0.478	*0.001*
Extensibility muscle-tendon complex (mm)	10.31 (2.12-15.54)	8.81 (4.31-13.84)	0.735	16.40 (12.50-22.58)	10.45 (7.86-15.00)	0.013	0.684	0.043
Work - high velocity (J)	3.10 (2.44-4.01)	2.75 (2.12-3.52)	0.510	2.45 (2.16-4.05)	1.90 (0.93-2.46)	*0.013*	0.050	0.077

Work - low velocity (J)	1.30 (0.80-2.14)	1.63 (0.50-3.02)	0.433	1.53 (1.05-1.99)	1.45 (0.22-2.51)	0.875	0.946	1.000
rmsEMG MG - high velocity (μV)	11.70 (7.30-21.90)	5.71 (2.55-23.97)	0.133	7.62 (5.72-10.30)	2.10 (0.63-4.30)	*0.003*	*0.014*	0.650
rmsEMG LG - high velocity (μV)	6.06 (3.64-13.24)	4.27 (1.84-9.63)	*0.047*	4.57 (1.54-6.38)	1.88 (0.78-6.79)	0.859	0.283	0.315
rmsEMG SOL - high velocity (μV)	5.50 (2.02-12.80)	6.46 (1.23-9.46)	0.034	4.83 (2.44-7.34)	8.03 (2.80-10.63)	0.657	0.611	0.169
Angular velocity – low velocity (deg/s)	21.75 (14.38 – 24.86)	17.98 (15.00 – 23.00)	0.507	20.12 (17.31 – 25.91)	19.48 (15.81 – 24.78)	0.875	0.550	0.454
Angular velocity – high velocity (deg/s)	167.46 (149.35 – 223.28)	170.33 (147.96 – 199.47)	0.345	174.91 (155.79 – 208.33)	173.94 (155.69 – 193.79)	0.826	0.756	0.603
ROM (degrees)	60.47 (55.45-70.26)	62.82 (52.16-69.08)	0.594	61.02 (45.33-74.42)	55.71 (47.64-70.25)	0.096	0.352	0.571

maxDF = maximum dorsiflexion, ML = muscle length, TL = tendon length, mm = millimeter, J = joule, rmsEMG = root mean square of the electromyographic signal MG = medial gastrocnemius muscle, LG = lateral gastrocnemius muscle, μV = microvolt, SOL = soleus muscle, ROM = range of motion

Supplementary Table III Results when only including subjects who had both ultrasound and Instrumented assessment of ankle joint resistance and stretch reflexes Highlighted p-values were found to be significant when analyzing the total groups (n=14 casting group; n=17 Botulinum NeuroToxin-A group).

Parameters that were found to be significant when analyzing the total groups	**Casting N=13**			**Botulinum NeuroToxin-A N=9**			**Between groups – post intervention**
	**PRE intervention**	**POST intervention**	**p-value**	**PRE intervention**	**POST intervention**	**p-value**	**p-value**
Ankle angle - resting (degrees)	-30.00 (-42.50 – -25.00)	-25.00 (-30.00 – -20.00)	0.004	-30.00 (-42.50 – -25.00)	-30.00 (-42.50 – -25.00)	0.739	
Ankle angle - maxDF (degrees)	0.00 (-7.50 – -7.50)	0.00 (0.00 – 10.00)	0.026	0.00 (-7.50 – 7.50)	0.00 (-7.50 – 7.50)	0.746	
ML - resting (mm)	132.00 (122.84 – 147.79)	132.18 (118.99 – 151.56)	0.959	132.00 (122.8 – 147.79)	132.18 (118.99 – 151.56)	0.069	0.082
TL - maxDF (mm)	137.30 (116.09 – 150.82)	145.42 (128.41 – 152.40)	0.039	137.30 (116.09 – 150.82)	145.42 (128.41 – 152.40)	0.161	
Muscle-tendon complex - maxDF (mm)	271.78 (252.36 – 304.70)	287.66 (266.74 – 316.30)	0.005	271.78 (252.36 – 304.70)	287.66 (266.74 – 316.36)	0.484	
Work - high velocity (J)	3.08 (2.29 – 4.04)	2.92 (1.93 – 3.67)	0.650	3.08 (2.29 – 4.04)	2.92 (1.93 – 3.67)	0.173	
rmsEMG MG - high velocity (μV)	14.03 (7.73 – 23.32)	7.45 (2.15 – 24.86)	0.099	14.03 (7.73 – 23.32)	7.50 (2.15 – 24.86)	0.021	0.049
rmsEMG LG - high velocity (μV)	6.07 (4.08 – 13.25)	4.68 (2.28 – 10.30)	0.066	6.07 (4.08 – 13.25)	4.68 (2.28 – 10.30)	0.144	
rmsEMG SOL - high velocity (μV)	6.84 (2.42 – 13.16)	6.58 (1.71 – 9.46)	0.050	6.84 (2.42 – 13.16)	6.58 (1.71 – 9.46)	1.000	

maxDF = maximum dorsiflexion, ML = muscle length, TL = tendon length, mm = millimeter, J = joule, rmsEMG = root mean square of the electromyographic signal MG = medial gastrocnemius muscle, LG = lateral gastrocnemius muscle, μV = microvolt, SOL = soleus muscle.

Supplementary Table IV. Correlations between changes in ultrasound and Instrumented assessment of ankle joint resistance and stretch reflex parameters of the casting group (n=13).

Casting

		Change in resting angle	Change in maxDF angle	Change in Δ ankle angle	Change in muscle-tendon complex length, rest	Change in muscle-tendon complex length, maxDF	Change in ML, rest	Change in ML, maxDF	Change in TL, rest	Change in TL, maxDF	Change MG rmsEMG (μV)	Change LG rmsEMG (μV)	Change SOL rmsEMG (μV)	Change ROM	Change work, low velocity	Change work, high minus low velocity	Change extensibility ML	Change extensibility TL
change in maxDF angle	Correlation Coefficient	0,076																
	Sig. (2-tailed)	0,806	.															
change in Δ ankle angle	Correlation Coefficient	-,703**	0,511															
	Sig. (2-tailed)	0,007	0,075	.														
Change in muscle-tendon complex length, rest	Correlation Coefficient	-0,443	-0,35	0,24														
	Sig. (2-tailed)	0,199	0,321	0,504	.													
Change in muscle-tendon complex length, maxDF	Correlation Coefficient	-0,205	0,558	0,419	-0,214													
	Sig. (2-tailed)	0,57	0,094	0,228	0,645	.												
Change in ML, rest	Correlation Coefficient	-0,26	0,058	0,409	0,309	-0,679												
	Sig. (2-tailed)	0,469	0,873	0,241	0,385	0,094	.											
Change in ML, maxDF	Correlation Coefficient	0,466	-0,162	-0,306	-0,5	0,164	-0,393											
	Sig. (2-tailed)	0,175	0,654	0,389	0,253	0,651	0,383	.										
Change in TL, rest	Correlation Coefficient	-0,188	0,081	0,212	,709*	0,152	-0,212	-,636*										
	Sig. (2-tailed)	0,539	0,791	0,486	0,022	0,676	0,556	0,048										
Change in TL, maxDF	Correlation Coefficient	-0,549	0,172	,555*	0,333	,685*	0,091	-0,588	,599*									
	Sig. (2-tailed)	0,052	0,575	0,049	0,347	0,029	0,803	0,074	0,031									
Change MG rmsEMG (μV)	Correlation Coefficient	0,244	0,519	0,156	0,317	0,079	-0,067	-0,079	0,259	-0,119								
	Sig. (2-tailed)	0,445	0,084	0,628	0,406	0,829	0,865	0,829	0,417	0,713								
Change LG rmsEMG (μV)	Correlation Coefficient	,740*	0,289	-0,235	-,821*	-0,107	-0,357	0,643	-0,583	-,667*	0,048							
	Sig. (2-tailed)	0,023	0,45	0,543	0,023	0,819	0,432	0,119	0,099	0,05	0,911	.						
Change SOL rmsEMG (μV)	Correlation Coefficient	0,014	-0,096	-0,181	0,467	0,071	-0,167	-0,333	0,473	0,027	0,115	-0,5						
	Sig. (2-tailed)	0,967	0,778	0,594	0,205	0,867	0,668	0,42	0,142	0,937	0,751	0,207	.					
Change ROM	Correlation Coefficient	0,266	-0,55	-,620*	0,03	-0,321	0,285	0,006	-0,11	0	-0,357	-0,017	-0,073					
	Sig. (2-tailed)	0,379	0,052	0,024	0,934	0,365	0,425	0,987	0,721	1	0,255	0,966	0,832					
Change work, low velocity	Correlation Coefficient	,756**	0,192	n.a.	-0,115	0,176	-0,491	0,273	0,11	-0,28	0,315	0,6	0,445					
	Sig. (2-tailed)	0,003	0,53	n.a.	0,751	0,627	0,15	0,446	0,721	0,354	0,319	0,088	0,17					
Change work, high minus low velocity	Correlation Coefficient	,762**	-0,157	n.a.	-0,503	-0,345	-0,248	0,248	-0,088	-,599*	-0,056	0,55	0,173		,555*			
	Sig. (2-tailed)	0,002	0,608	n.a.	0,138	0,328	0,489	0,489	0,775	0,031	0,863	0,125	0,612		0,049			
Change extensibility ML	Correlation Coefficient	0,501	0,039	-0,335	-0,75	0,607	-,821*	0,714	-0,179	-0,143	-0,357	0,6	-0,314	-0,143	0,393	0,429		
	Sig. (2-tailed)	0,252	0,933	0,463	0,052	0,148	0,023	0,071	0,702	0,76	0,432	0,285	0,544	0,76	0,383	0,337		
Change extensibility TL	Correlation Coefficient	-0,364	-0,099	0,229	-0,297	0,273	0,285	0,503	-,714**	0,044	-0,357	-0,033	-,618*	0,033	-0,451	-0,467	0,214	
	Sig. (2-tailed)	0,221	0,748	0,451	0,405	0,446	0,425	0,138	0,006	0,887	0,255	0,932	0,043	0,915	0,122	0,108	0,645	
Change extensibility muscle-tendon complex	Correlation Coefficient	0,309	0,079	-0,256	-,857*	0,536	-0,607	0,536	-0,429	0,071	-0,679	0,6	-0,771	0,143	0,036	0,25	,857*	0,536
	Sig. (2-tailed)	0,501	0,867	0,579	0,014	0,215	0,148	0,215	0,337	0,879	0,094	0,285	0,072	0,76	0,939	0,589	0,014	0,215

maxDF = maximum dorsiflexion, ML = muscle length, TL = tendon length, mm = millimeter, J = joule, rmsEMG = root mean square of the electromyographic signal MG = medial gastrocnemius muscle, LG = lateral gastrocnemius muscle, μV = microvolt, SOL = soleus muscle, ROM = range of motion.* Correlation is significant at the 0.05 level (2-tailed). ** Correlation is significant at the 0.01 level (2-tailed).

Supplementary Table V. Correlations between changes in ultrasound and Instrumented assessment of ankle joint resistance and stretch reflex parameters of the BoNT-A group (n=9).

BoNT-A

		Change in resting angle	Change in maxDF angle	Change in Δ ankle angle	Change in muscle-tendon complex length, rest	Change in muscle-tendon complex length, maxDF	Change in ML, rest	Change in ML, maxDF	Change in TL, rest	Change in TL, maxDF	Change MG rmsEMG (μV)	Change LG rmsEMG (μV)	Change SOL rmsEMG (μV)	Change ROM	Change work, low velocity	Change work, high minus low velocity	Change extensibility ML	Change extensibility TL
change in maxDF angle	Correlation Coefficient	-0,424																
	Sig. (2-tailed)	0,255	.															
change in Δ ankle angle	Correlation Coefficient	-,673*	,924**															
	Sig. (2-tailed)	0,047	0	.														
Change in muscle-tendon complex length, rest	Correlation Coefficient	0,025	0,037	0,05														
	Sig. (2-tailed)	0,953	0,931	0,906	.													
Change in muscle-tendon complex length, maxDF	Correlation Coefficient	0,025	0,63	0,43	,857*													
	Sig. (2-tailed)	0,954	0,094	0,288	0,014	.												
Change in ML, rest	Correlation Coefficient	0,05	0,482	0,401	,738*	,786*												
	Sig. (2-tailed)	0,906	0,227	0,325	0,037	0,036	.											
Change in ML, maxDF	Correlation Coefficient	-0,477	0,472	0,552	,833*	0,643	,714*											
	Sig. (2-tailed)	0,195	0,199	0,123	0,01	0,086	0,047	.										
Change in TL, rest	Correlation Coefficient	-0,121	-0,232	-0,104	0,143	-0,048	-0,5	0,25										
	Sig. (2-tailed)	0,756	0,548	0,791	0,736	0,911	0,207	0,516	.									
Change in TL, maxDF	Correlation Coefficient	0,42	-0,012	-0,16	0,214	0,357	0,321	-0,429	-0,381									
	Sig. (2-tailed)	0,3	0,977	0,706	0,645	0,385	0,482	0,289	0,352	.								
Change MG rmsEMG (μV)	Correlation Coefficient	-0,191	-0,086	0,155	-0,119	-0,286	-0,333	0,117	0,6	-0,143								
	Sig. (2-tailed)	0,623	0,826	0,69	0,779	0,493	0,42	0,765	0,088	0,736	.							
Change LG rmsEMG (μV)	Correlation Coefficient	0,949	-0,2	-0,316	-0,4	-0,2	-0,4	-0,4	0,8	-0,4	0,8							
	Sig. (2-tailed)	0,051	0,8	0,684	0,6	0,8	0,6	0,6	0,2	0,6	0,2	.						
Change SOL rmsEMG (μV)	Correlation Coefficient	0,113	0,692	0,543	0,357	0,679	,786*	0,286	-0,643	0,607	-0,5	-0,4						
	Sig. (2-tailed)	0,809	0,085	0,208	0,432	0,094	0,036	0,535	0,119	0,148	0,253	0,6	.					
Change ROM	Correlation Coefficient	-0,269	0,129	0,069	-0,119	-0,119	0,095	-0,183	-,667*	0,048	-0,533	-0,8	-0,036					
	Sig. (2-tailed)	0,485	0,741	0,86	0,779	0,779	0,823	0,637	0,05	0,911	0,139	0,2	0,939	.				
Change work, low velocity	Correlation Coefficient	-0,529	0,532	,690*	0,381	0,452	0,143	0,65	0,5	-0,214	0,4	0,2	0,214	-0,483				
	Sig. (2-tailed)	0,143	0,14	0,04	0,352	0,26	0,736	0,058	0,17	0,61	0,286	0,8	0,645	0,187	.			
Change work, high minus low velocity	Correlation Coefficient	0,477	-0,309	-0,38	-0,476	-0,167	-0,619	-,767*	0	0,5	-0,083	0,4	-0,071	-0,167	-0,133			
	Sig. (2-tailed)	0,195	0,418	0,314	0,233	0,693	0,102	0,016	1	0,207	0,831	0,6	0,879	0,668	0,732	.		
Change extensibility ML	Correlation Coefficient	-0,202	-0,469	-0,225	0,143	-0,393	-0,452	0,167	,762*	-0,536	0,595	1,000**	-,857*	-0,19	0,381	0,071		
	Sig. (2-tailed)	0,632	0,241	0,592	0,736	0,383	0,26	0,693	0,028	0,215	0,12	.	0,014	0,651	0,352	0,867	.	
Change extensibility TL	Correlation Coefficient	0,408	0,025	-0,147	0,393	0,333	0,714	-0,238	-0,69	,786*	-0,643	-0,8	,929**	0,19	-0,31	0,286	-,893**	
	Sig. (2-tailed)	0,316	0,954	0,728	0,383	0,42	0,071	0,57	0,058	0,021	0,086	0,2	0,003	0,651	0,456	0,493	0,007	.
Change extensibility muscle-tendon complex	Correlation Coefficient	0,094	0,505	0,393	0,429	0,714	0,5	0,036	-0,321	,929**	-0,107	-0,4	,786*	-0,071	0,321	0,286	-0,571	,821*
	Sig. (2-tailed)	0,84	0,247	0,383	0,337	0,071	0,253	0,939	0,482	0,003	0,819	0,6	0,036	0,879	0,482	0,535	0,18	0,023

maxDF = maximum dorsiflexion, ML = muscle length, TL = tendon length, mm = millimeter, J = joule, rmsEMG = root mean square of the electromyographic signal MG = medial gastrocnemius muscle, LG = lateral gastrocnemius muscle, μV = microvolt, SOL = soleus muscle, ROM = range of motion. * Correlation is significant at the 0.05 level (2-tailed). ** Correlation is significant at the 0.01 level (2-tailed).

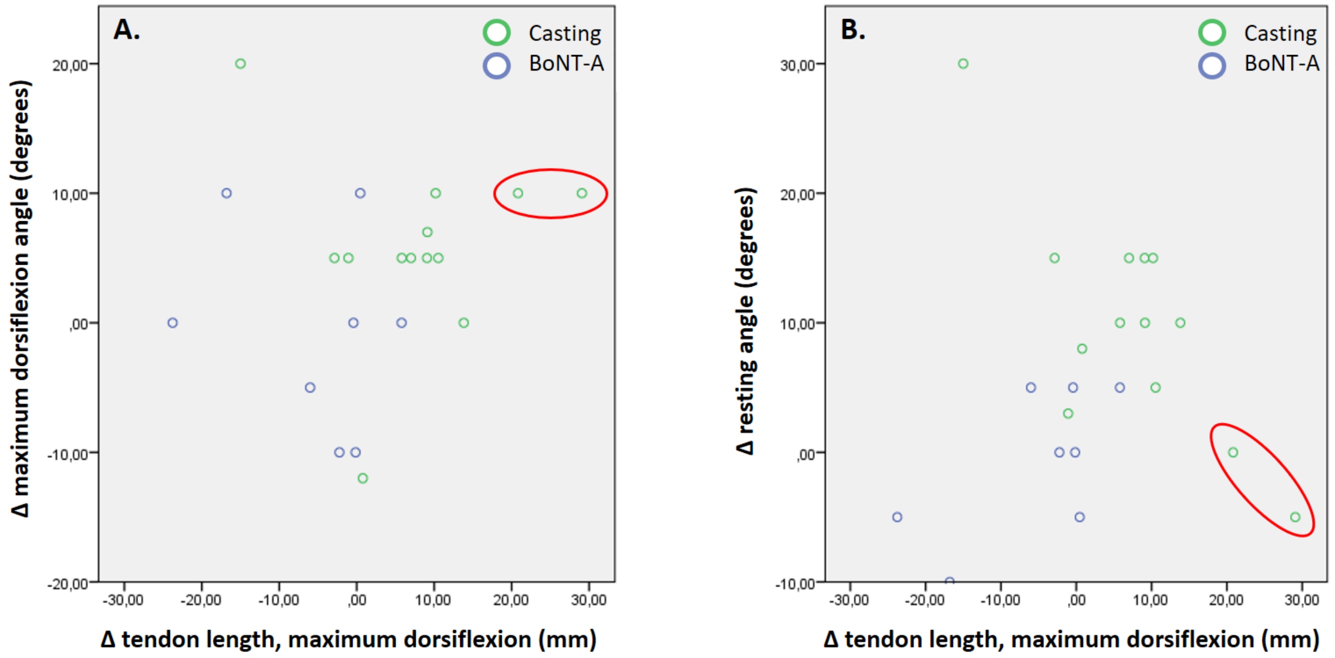

Supplementary Figure I. Scatterplots of change (post-pre) in tendon length at maximum dorsiflexion and change (post-pre) in (A) maximum dorsiflexion angle and (B) change (post-pre) in resting angle. Highlighting the deviating response of two subjects in the casting group.

Note that in figure A. those two subjects whose maximum dorsiflexion angle increased by 10deg without (or minimal) change in the resting angle, there was also a corresponding increase in tendon length.
